# Vitamin D and the risk of latent tuberculosis infection: a systematic review and meta-analysis

**DOI:** 10.1186/s12890-022-01830-5

**Published:** 2022-01-19

**Authors:** Yan Cao, Xinjing Wang, Ping Liu, Yue Su, Haotian Yu, Jingli Du

**Affiliations:** 1 Tuberculosis Department, The 8Th Medical Center of Chinese, PLA General Hospital, Beijing, China; 2 The 8Th Medical Center of Chinese, PLA General Hospital, Beijing, China; 3 The Second Medical Center and National Clinical Research Center for Geriatric Diseases, Chinese PLA General Hospital, Beijing, China

**Keywords:** Latent tuberculosis infection, Vitamin D, 25(OH)D, Meta-analysis

## Abstract

**Objective:**

Latent tuberculosis infection (LTBI) may be a risk of developing tuberculosis (TB) and thus a health hazard. The aim of this meta-analysis is to explore the association between vitamin D and LTBI.

**Methods:**

Databases including PubMed, Embase, Scopus, and ProQuest were electronically searched to identify observational or interventional studies that reported the association between vitamin D and LTBI. The retrieval time is limited from inception to 30 September 2021. Two reviewers independently screened literature, extracted data, and assessed risk bias of included studies. Meta-analysis was performed by using STATA 12.0 software.

**Results:**

A total of 5 studies involving 2 case–control studies and 3 cohort studies were included. The meta-analysis result showed that the risk of LTBI among individuals was not associated with high vitamin D level (OR 0.51, 95% CI 0.05–5.65, *P* = 0.58). The result from cohort studies also suggested that relatively high vitamin D level was not a protective factor for LTBI (RR = 0.56, 95%CI 0.19–1.67, *P* = 0.300).

**Conclusions:**

Our meta-analysis suggested that serum vitamin D levels were not associated with incidence of LTBI, and relatively high serum vitamin D level was not a protective factor for LTBI. Further RCTs are needed to verify whether sufficient vitamin D levels and vitamin D supplementation reduces the risk of LTBI.

**Supplementary Information:**

The online version contains supplementary material available at 10.1186/s12890-022-01830-5.

## Introduction

Tuberculosis (TB) is a major global public health crisis, resulting in 1.5 million deaths in 2018 [1]. It is estimated that a quarter of the world’s population is infected with *Mycobacterium tuberculosis* globally in 2014, and thus may be a risk of developing TB, especially soon after infection [2]. Latent TB infection (LTBI) is a state in which *Mycobacterium tuberculosis* survives in a dormant condition in the host, and individuals infected with LTBI have neither symptoms nor infectivity [3]. Approximately 5–15% of LTBI infected will develop active tuberculosis in their lifetime [4, 5]. Multiple studies have reported that a considerable proportion of TB cases were caused by the progression of LTBI [6, 7]. Therefore, tackling LTBI will be a crucial priority as the large number of asymptomatic infected people threaten the elimination of TB [8].

It has been reported that various factors may influence the incidence and progression of TB, one of which was vitamin D deficiency [9–11]. In addition to playing a major role in bone metabolism, vitamin D is also important in preventing infection [12]. Several studies have found that 1,25-dihydroxyvitamin D (1,25(OH)_2_D_3_), which is the bioactive form of vitamin D, binds to the vitamin D receptor (VDR), activates the VDR signaling and induces a series of antimicrobial responses, such as inducing autophagy, activation of antimicrobial peptides, and killing intracellular *Mycobacterium tuberculosis* [13–15]. In order to study whether vitamin D was associated with tuberculosis, numerous studies have been conducted. However, only few studies have reported the association between vitamin D and LTBI, and they produced inconsistent and varying results [16, 17].

In view of the previous lack of meta-analysis to explore the relationship between vitamin D status or intake and LTBI, this meta-analysis was conducted for patients with LTBI to explore whether the vitamin D supplementation or high level of serum vitamin D is associated with the decrease of the risk of LTBI, which may provide nutritional support for the prevention of tuberculosis.

## Materials and methods

Our study does not require ethical approval and informed consent because it is a systematic review and meta-analysis of previously published literature and does not address ethics or patient privacy. Our study was analyzed and reported according to the Preferred Reporting Items for Systematic Reviews and Meta-Analyses (PRISMA) [18]. PRISMA flow diagram is used for displaying the selection of articles from the search.

### Protocol and registration

This analysis was not registered. A review protocol does not exist for this analysis.

### Data sources and search strategy

A systematic search of PubMed, Embase, Scopus, and ProQuest was performed from inception to 30 November 2021 for studies evaluating the efficacy of Vitamin D in the risks of LTBI. The search strategy used subject headings and keywords without language and date restrictions. Additional file 1 provides details of our search strategy. We searched PROSPERO to confirm that no other similar study was conducted in progress simultaneously. Manual reference checks were performed pertinent studies to determine further relevant trials. Any differences in the study selection process were resolved through panel discussions (CY, WXJ, LP and SY).

### Criteria for study selection

Studies meeting the following criteria were included in the final analysis: All types of prospective studies (including RCTs, cohort study or case–control study, etc.) that reported on the association between Vitamin D and the risks of LTBI in children or adults. Studies were excluded if: (a) did not report the risks of LTBI or Vitamin D; (b) were conference reports, case reports, reviews or letters; (c) did not report the results of odds ratio(OR), relative risk (RR) or their 95% confidence interval (CI); (d) measured vitamin D levels among individuals already diagnosed with TB disease or latent TB infected.

### Data extraction

Two reviewers independently evaluated the titles and abstracts of all articles retrieved in the initial search between the two reviewers. A screen form was used to exclude articles that did not meet the eligibility criteria with a hierarchical approach based on study design, population or exposure and outcome. If the article was deemed to be eligible, the full-text was retrieved and its relevance was evaluated by two of the authors (CY, WXJ). Any disagreements are discussed and resolved by consensus.

Two independent reviewers extracted data of selected studies separately using a standard data extraction form. Extracted information included: study details (name of the first author, publication year, location, study design, total number of participants and cases), population characteristics (description of the included study population, mean or median age and their standard deviation (SD) or inter-quartile range (IQR), number and percentage of female), exposure (method of measuring Vitamin D, mean/median baseline 25-OH Vitamin D, length of follow-up, and LTBI definition), and outcomes (estimate and their 95% CI, such as OR and RR) and adjusted for confounders. Any discrepancies in data extraction were discussed and assessed by a third reviewer for resolution from 2 December 2021 to 3 December2021.

### Quality assessment

Case–control and cohort studies were assessed using the Newcastle–Ottawa scale (NOS) [19] consisting of three domains: (1) selection of subjects, (2) comparability of groups, and (3) assessment of outcome. A score of 0–9 was allocated to each relevant study. While the NOS has no established thresholds, we considered the quality of each study as low (0–3), moderate (4–6), or high (7–9) [20]. When inconsistency exists, a third reviewer will make the final decision after verification and discussion.

### Statistical analysis

In our meta-analysis, OR, RR and their 95% CI were used as the effect measure. Heterogeneity was assessed by by Higgins’ I^2^ statistics(values range from 0 to 100%.) and random effect model was selected when heterogeneity was significant (I^2^ > 50%), otherwise, fixed effect model was chosen [21]. However, if the small number of studies were included in the review, I^2^ has a substantial bias and random effect model may be more appropriate [22]. In our main analysis, we divided the participants into two groups by vitamin D levels (30 ng/ml compared with < 30 ng/ml). The sample size of each subgroup and the target event were added to calculate the unajusted log (OR) or log (RR). The inverse variance method was used for Meta analysis after merging data. Forest plots were used to display the results from individual studies and pooled estimates, and *P* < 0.05 were regarded as statistically significant. Begg's and Egger's test were performed to evaluate potential publication bias [23]. Funnel plots were visually evaluated for asymmetry [24]. Sensitivity analysis was performed to evaluate the robustness of the results. All statistical analyses were performed with Stata 12.0 (StataCorp, College Station, TX, USA).

## Results

### Results of study search

**Fig. 1:**
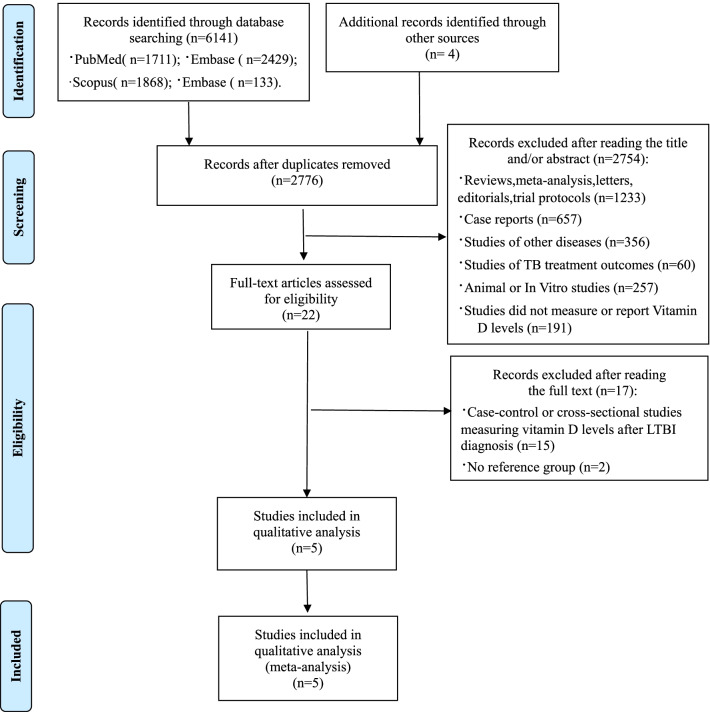
PRISMA chart: flow diagram of the process of selection of articles

A search strategy identified a total of 6145 articles published from database inception through 30 November 2021. Following electronic deduplication, one author (CY) reviewed all potentially duplicate records and removed 3369 true duplicates, resulting in a total of 2776 records. After screening titles and abstracts, we excluded 2754 articles because they were reviews, meta-analyses, letters, editorials or trial protocols (n = 1233), case reports (n = 657), studies of other diseases (n = 356), studies of TB treatment outcomes (n = 60), animal or in vitro studies (n = 257), studies did not measure or report Vitamin D levels (n = 191). We reviewed full texts of the remaining 22 articles and further excluded 15 studies that were case–control or cross-sectional studies that assessed vitamin D levels after LTBI diagnosis and 2 study of no reference group. Finally, a total of 5 articles [16, 17, 25–27] meeting the criteria for the quantitative synthesis were included in the meta-analysis (Fig. 1).

### Characteristics of the studies

Table 1 sets out the characteristics of the five included studies [16, 17, 25–27]. There were 3 cohort studies [16, 17, 27], and 2 case–control studies [25, 26]. Publication ranged from 2011 to 2020 and included two high tuberculosis burden countries, Brazil and Indonesia; and only one low tuberculosis burden country, Spain, predominating with three studies. One of the five studies was based on Brazilian prisoners [25], and other four studies were based on contacts of TB patients [16, 17, 26, 27], two of which including household members, family members, friends and work or study colleagues [16, 26]; one including Household case contacts [27]; and one conducted in five nursing homes and one mental disability institution including residents, staff members and relatives of TB cases [17]. Overall, the selected 5 studies included 1516 subjects, comprising 117 LTBI patients for this meta-analysis. Diagnosis of LTBI in all studies was based upon QFN-GIT, IGRAs and/or TST. One study assessed serum 25–(OH)D levels using ID-LC–MS/MS [27], whereas others used ECLIA [25, 26] and CLIA [16, 17]. 3 articles reported the outcome of RR and 95%CI, 2 articles used OR as outcome. The included studies reported the association between vitamin D and latent tuberculosis infection. 2 case–control and 3 cohort studies were considered as high quality, because the study design had been described in detail (Tables 2, 3).

**Table 1:** Summary of studies included in the meta-analysis

Study [Reference]	Country	Study design	Population	Total number of participants	Number of LTBI cases	Median/Mean age, years (IQR/SD)	Female, n (%)	Method of Measuring Vitamin D	Median baseline 25-OH Vitamin D, nmol/L(ng/ml) (IQR/SD)	Length of follow- up	LTBI disease definition	Effect estimate (95% CI)
Maceda 2018 [24]	Brazil	Nested case–control study	Over 18 years old Brazilian prisoners	90	30	Cases: 32.1 (6.87) Controls: 31.1 (7.71)	NR	ECLIA	Cases: 37.7 (11.93) Controls: 34.5 (14.89)	1 year	Smear and/or culture positive	OR for vitamin D and TST, ≥ 30 ng/ml compared with < 30 ng/ml: 1.27 (0.48–3.38)
Arnedo-Pena 2011 [25]	Spain	Cross-sectional and case–control study	Contacts of TB patients	93	11	Cases: 39.6 (13.1) Controls: 34.7 (14.5)	Cases: 3 (29.3) Controls: 41 (50.0)	ECLIA	Cases: 17.5 (5.6) Controls: 25.9 (13.7)	2 months	The change from negative to positive TST with an increase of ≥ 5 mm on the induration in non BCG-vaccinated participants, and an increase of ≥ 10 mm on the induration from the initial TST, or the presence of vesicles in BCG-vaccinated participants	OR for vitamin D and TST, ≥ 30 ng/ml compared with < 30 ng/ml: 0.10 (0.01–1.73)
Arnedo-Pena 2020 [17]	Spain	Cross-sectional and prospective cohort study	Exposed population of TB cases	837	166	Cases: 79 (range 24–95) Non-cases: 56 (range 18–105)	Cases: 100 (60.2) Non-cases: 521 (77.6)	CLIA	Cases: 18.6 (13.4) Non-cases: 19.6 (12.8)	8–10 weeks	QFT, interferon-gamma release assay or TST positive and chest radiography	RR for vitamin D and LTBI, ≥ 30 ng/ml compared with < 30 ng/ml: 0.48 (0.16–1.40)
Arnedo-Pena 2015 [16]	Spain	Prospective cohort study	Contacts of pulmonary TB patients	198	18	Cases: 32.8 (17.2) Non-cases: 38.1 (12.4)	Cases: 6 (33.3) Non-cases: 92 (51.1)	CLIA	Cases: 20.7 (11.9) Non-cases: 27.2 (11.4)	8–10 weeks	A change from negative QFT-GIT in the first test to positive QFT-GI in the second test with an increase of at least 2.6 times the first QFT-GIT test (LTBI); and thorax radiology	a RR for vitamin D and LTBI, ≥ 30 ng/ml compared with < 30 ng/ml: 0.22 (0.07–0.70)
Verrall 2017 [26]	Indonesia	Prospective cohort study	Household case contacts	298	76	Cases: 40 (15) Non-cases: 40 (15)	Cases: 110 (49.5) Non-cases: 43 (56.6)	ID-LC–MS/MS	54.1 (21.5) nmol/L	14 weeks	Quantiferon Gold In Tube (QFN-GIT) tested positive (LTBI)	a RR for vitamin D and LTBI, ≥ 30 ng/ml compared with < 30 ng/ml: 1.29 (0.73–2.30)

*Abbreviations*: LTBI, latent tuberculosis infection; QFT, QuantiFERON-TB Gold In-Tube assay; TST, tuberculin skin test; ART, antiretroviral therapy; TBIC, tuberculosis infection conversion; ECLIA, electrochemiluminescence immunoassay; CLIA, chemiluminescence immunoassay; ID-LC–MS/MS, isotope-dilution liquid chromatography tandem mass spectroscopy

**Table 2:** Case–control studies bias assessment using the Newcastle–Ottawa Scale (NOS)

Study	Selection				Comparability	Exposure			Total score
	Is the case definition adequate	Representativeness of the cases	Selection of controls	Definition of controls	Comparability of cases and controls of design or analysis	Ascertainment of exposure	Same method of ascertainment for cases	Non-response rate	
Maceda 2018 [24]	1	1	1	1	1	1	1	0	7
Arnedo-Pena 2011 [25]	1	1	1	1	1	1	1	0	7

**Table 3:** Cohort studies bias assessment using the Newcastle–Ottawa Scale (NOS)

Study	Selection				Comparability		Assessment of outcome			Total score
	Representativeness of exposure arm(s)	Selection of the comparative arm(s)	Origin of exposure source	Demonstration that outcome of interest was not present at start of study	Studies controlling the most important factors	Studies controlling the other main factors	Assessment of outcome with independency	Adequacy of follow-up length	Lost to follow-up acceptable	
Arnedo-Pena 2020 [17]	1	1	1	1	1	0	1	1	1	8
Arnedo-Pena 2015 [16]	1	1	1	1	1	0	1	1	0	7
Verrall 2017 [26]	1	1	1	1	1	1	1	1	1	9

### Vitamin D level was associated with the risk of LTBI

**Fig. 2:**
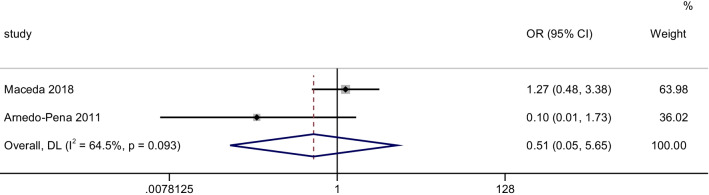
Forest plots for the association between Vitamin D levels and the risk of LTBI: overall effect for dichotomous outcome using a fixed-effect model. The diamonds stand for pooled effect

A total of 2 case–control studies with 41 LTBI cases and 142 control subjects were included in our analysis. Our analysis with a random effect model (inverse-variance) showed that relatively high serum Vitamin D level was not significantly associated with a decreased risk of LTBI (OR = 0.51, 95% CI 0.05–5.65, *P* = 0.58, I^2^ = 64.5%) (Fig. 2).

### Vitamin D was more likely a risk factor for LTBI than its consequence

**Fig. 3:**
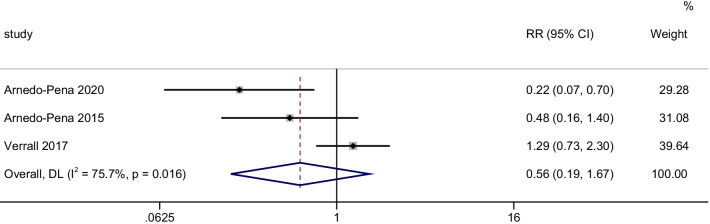
Forest plots for the association between Vitamin D levels and the incidence of LTBI: overall effect for dichotomous outcome using a fixed-effect model. The diamonds stand for pooled effect

3 cohort studies involving 1333 participants were included in our meta-analysis. Our analysis with a random effect model (inverse-variance)showed relatively high serum vitamin D levels can′t reduce the incidence of LTBI (RR = 0.56, 95% CI 0.19–1.67, *P* = 0.300, I^2^ = 75.7%) (Fig. 3).

### Between-studies heterogeneity and publication bias

Between-studies heterogeneity varied from none to small for our meta-analyses with I^2^ values ranging from 64.5 to 75.7%. However, sensitivity analysis and visual inspection of funnel plots for checking asymmetry and publication bias weren’t done because only 2 articles were included in association of vitamin D and the risk of LTBI and 3 in association of vitamin D and the incidence of LTBI.

## Discussion

The present meta-analysis of case–control studies showed that the relatively high serum vitamin D level was not associated with the decreased risk of LTBI. The result of cohort studies suggested that the decreased incidence of LTBI was not associated with the elevated serum vitamin D levels.

Although some previous studies have documented poor vitamin D status among patients with active TB compared to healthy controls [28–30], relatively few studies have prospectively investigated the role of preexisting vitamin D level in the LTBI prevention. Our findings do not provide evidence for subnormal vitamin D increasing susceptibility to TB infection. Experimental studies show 25-OH vitamin D modulates the innate response to infection, including the production of antimicrobial peptides, and cytokine production. After entering into the human body, *Mycobacterium tuberculosis* will be lysed in the phagosomes inside macrophages. If the concentration of Ca^2+^ in cells does not increase, *Mycobacterium tuberculosis* resides in phagosomes are not lysed, thereby resulting in LTBI [31]. LTBI patients may develop TB in the near or distant future [32]. Several case–control studies have linked vitamin D deficiency to active disease, although their interpretation is complicated by potential reverse causality [33]. Despite this, clinical studies have not revealed a role for vitamin D in treating active disease. In addition, the effects of 1,25-(OH)_2_D on *Mycobacterium tuberculosis* infection are complex and have not been described in detail in previous studies [11, 34]. Overall there was no significant association between serum vitamin D levels and incidence of LTBI. However, the confidence intervals for the effect of relatively high serum Vitamin D level were wide, meaning the present study does not exclude the possibility that risk of TB infection is almost reduced by half in sufficient vitamin D. Maceda [25] and Verral [27] found that low levels of vitamin D may be not associated with risk of M. tuberculosis infection. We considered possible explanations for why they did not detect a significant association between vitamin D levels and LTBI risk. Firstly, in the individuals at enrollment, both diet and sun exposures appear to be adequate to sustain precursor vitamin D levels in healthy subjects. It is possible that there was insuffcient heterogeneity in vitamin D in this population and setting to detect such effects. Secondly, these studies have been found to vary with study design, geographical location, as well as genders and follow-up periods of subjects. Thirdly, We also note that the different 25-(OH)D assays employed in the meta-analysis studies vary in their sensitivity and precision. Thus, studies with larger sample sizes in different populations are needed to help clarify whether the association between vitamin D and susceptibility to TB infection.

The hypothesis that vitamin D supplementation can prevent TB infection and progression from LTBI to TB disease has not been systematically reviewed. This is due to the lack of relevant studies [35]. Arnedo-pena et al. found that low plasma vitamin D was associated with tuberculin skin test (TST) positive conversion in a small number of contacts at follow-up [26]. Moreover, Gibney et al. observed that higher vitamin D levels were associated with lower LTBI prevalence among sub-Saharan African migrants in Melbourne, Australia [36]. According to previous reports, the vitamin D levels of LTBI patients were significantly lower than that of healthy people [36], the general population receiving vitamin D showed enhanced anti-TB immunity compared with those receiving placebo [37], and the TST conversion rate was lower in school children who received vitamin D, and their height increased [38]. Only a few studies have evaluated the role of vitamin D supplementation in preventing LTBI acquisition in contacts. A randomized controlled trial (RCT) by Martineau et al. found that compared with placebo group, the innate immunity against mycobacteria in vitamin D group was significantly improved, which was shown by recombinant Mycobacterium growth restriction (BCG-lux analysis), but the parameters of acquired immune response were not improved [37]. Thus, we can hypothesize that vitamin D may inhibit the progression from LTBI to active TB [39, 40].

One of the limitations of our study is that it is still not resolved whether vitamin D supplementation is conductive to the prevention of LITB. Previous meta-analysis found that vitamin D deficiency was associated with an increased risk of developing active TB in those subjects with LTBI and with an increased risk of TST conversion/TB infection conversion [41]. However, the meta-analysis did not address the question of whether vitamin D supplementation would be beneficial to LTBI prevention, and our study focused on the effect of higher serum vitamin D levels relative to lower vitamin levels on LTBI, rather than merely vitamin D deficiency, which is different from the previous meta-analysis. In addition, since all the studies included in the meta-analysis were observational studies and RCTs of vitamin D supplementation were few (only two articles were retrieved), we could only acquire the relationship between serum vitamin D level (25(OH)D) and LTBI. We can only assume that vitamin D supplementation prevents the activation of latent TB, and further RCTs are needed to verify this hypothesis. Furthermore, single-factor analyses of the included studies showed statistical differences in overall comparisons of multiple vitamin D levels, but did not specify which differences existed between the two groups and may be related to statistical differences between the maximum and minimum doses. However, when combining the effect size, the group with lower than normal level (< 30 ng/ mL) may weaken the difference between the whole group. Further multi-center, large-sample RCT need to be conducted to investigate whether sufficient vitamin D levels and vitamin D supplementation prevents LTBI.

## Conclusion

Our meta-analysis suggested that serum vitamin D levels were not associated with incidence of LTBI, and relatively high serum vitamin D level was not a protective factor for LTBI. Further RCTs are needed to verify whether sufficient vitamin D levels and vitamin D supplementation prevents LTBI.

## Supplementary Information

**Additional file 1**. Search strategy of vitamind D and the risk of latent tuberculosis infection.

## Data Availability

We used the data from published data given its nature of systematic review and meta-analysis.
