# Supplementary material for: Vitamin D and the risk of latent tuberculosis infection: a systematic review and meta-analysis
Source: BMC Pulm Med. 2022 Jan 19;22:39. doi: 10.1186/s12890-022-01830-5 (PMC8772077; doi:10.1186/s12890-022-01830-5)
Supplement: Supplementary file 1 — Additional file 1. Search strategy of vitamind D and the risk of latent tuberculosis infection. [file 12890_2022_1830_MOESM1_ESM.docx]

**Vitamin D and the risk of latent tuberculosis infection: a systematic review and meta-analysis**

**Supplement 1: search strategy**

Before 30 November 2021.

1. **Search strategy of Pubmed: 1711**

**Mesh Terms: 644**

((((tuberculosis[MeSH Terms]) OR (tuberculoses[MeSH Terms])) OR (Koch Disease[MeSH Terms])) OR (latent tuberculosis[MeSH Terms])) AND (((Vitamin D[MeSH Terms]) OR (25 (OH) vitamin D[MeSH Terms])) OR (25-hydroxyvitamin D[MeSH Terms]))

OR

**Text Terms:1067**

((((tuberculosis[Text Word]) OR (tuberculoses[Text Word])) OR (Koch Disease[Text Word])) OR (latent tuberculosis[Text Word])) AND (((Vitamin D[Text Word]) OR (25 (OH) vitamin D[Text Word])) OR (25-hydroxyvitamin D[Text Word]))

1. **Search strategy of Embase** **: 2429**

#1.’tuberculosis’/exp

#2.’tuberculosis’/:ab,ti

#3. ’latenet tuberculosis’/exp

#4. ’latenet tuberculosis’/:ab,ti

#5. #1 OR #2 OR #3 OR #4

#6 ‘vitamin d’/.exp

#7 ‘calcitriol’/exp

#8 ‘25 hydroxyvitamin d’/exp

#9 ‘vitamin AND d:ab,ti

#10 ‘calcitriol:ab,ti

#11 ‘25 hydroxyvitamin’ AND d:ab,ti

#12 #6 OR #7 OR #8 OR #9 OR #10 OR #11

#13 #5 AND #12

1. **Search strategy of Scopus** **: 1868**

TITLE-ABS-KEY ( "Vitamin D"  OR  "25 (OH) vitamin D"  OR  "25-hydroxyvitaminD" )

AND  TITLE-ABS-KEY ( "tuberculosis"  OR  "tuberculoses"  OR  "Koch Disease"  OR  "latent tuberculosis" )

1. **Search strategy of ProQuest:133**

(ab(vitamine d) OR ab(25 (OH) vitamin D) OR ab(25-hydroxyvitamin D)) AND (ab(tuberculosis) OR ab(tuberculoses) OR ab(Koch Disease) OR ab(latent tuberculosis))
